# Pattern of weight gain in pregnant women in slum areas of Hamadan using multilevel ordinal regression

**DOI:** 10.1186/s12889-023-15090-3

**Published:** 2023-01-27

**Authors:** Zohreh Manoochehri, Abbas Moghimbeigi, Khadije Ezzati-Rastegar, Javad Faradmal

**Affiliations:** 1 Department of Biostatistics, Student Research Committee, Hamadan University of Medical Sciences, Hamadan, Iran; 2 Department of Biostatistics and Epidemiology, Faculty of Health & Health, Safety and Environment Research Center, Alborz University of Medical Sciences, Karaj, Iran; 3 Department of Public Health, School of Public Health, Hamadan University of Medical Sciences, Hamadan, Iran; 4 Modeling of Noncommunicable Diseases Research Center, Department of Biostatistics, School of Public Health, Hamadan University of Medical Sciences, Hamadan, Iran

**Keywords:** Gestational weight gain, Slum areas, Multilevel ordinal models

## Abstract

**Background:**

Adequate gestational weight gain (GWG) is an important factor for maternal and fetal health. This is especially important in low-income and slum areas due to limited access to health services and malnutrition. Thus, the purpose of this study is to evaluate the pattern of GWG changes in the slum areas of Hamadan in Iran.

**Methods:**

In this longitudinal study, the study sample consisted of 509 pregnant women who referred to nine health care clinics in the slum areas of Hamadan. Women's weight gain based on the recommended GWG by U.S. Institute of Medicine (IOM) was divided into three categories: Inadequate weight gain, Adequate weight gain, and Excessive weight gain. In order to evaluate the trend of GWG, a multi-level ordinal model was used.

**Results:**

According to pre-pregnancy BMI, a little more than half people (56.6%) were overweight or obese. 85.4% women in the first trimester and 49.1% in the second trimester did not have adequate GWG, but in the third trimester (38.9%) had adequate GWG. Based on multivariate analysis, pre- pregnancy BMI has a significant effect on the odds of inadequate GWG (*P*-value = 0.021); with one unit increase in pre-pregnancy BMI, the odds of inadequate GWG grows by 1.07 times compared to adequate and excessive GWG.

**Conclusions:**

In general, women did not have adequate weight gain in the first and second trimesters.Thus, designing appropriate interventions to achieve optimal GWG seems to be necessary in slums.

## Introduction

Gestational weight gain (GWG) is a unique and complex biological phenomenon to support normal fetal growth and development [1]. This phenomenon is caused by changes in the uterus and its contents such as placenta, amniotic fluid, fetus, as well as metabolic changes in the mother's body, including the accumulation of fluid and fat [2]. The U.S. Institute of Medicine (IOM) has recommended guidelines to help physicians in monitoring adequate GWG [3]. According to these guidelines, the recommended GWG for different groups of pregnant women according to the pre-pregnancy body mass index is different (they will be presented later in Table 1) [4].

**Table 1:** recommendations for total and rate of weight gain during pregnancy, by pre-pregnancy BMI

Pre-pregnancy BMI (kg/m^2^)	Category of Pre –pregnancy BMI	Total weight gain range (kg)	Rates of weight gain from the beginning of week 13 onwards (kg / week)
< 18.5	under weight	12.5–18	0.51 (0.44–0.58)
18.5–24.9	normal weight	11.5–16	0.42 (0.35–0.50)
25–29.9	over weight	7–11.5	0.28 (0.23–0.33)
≥ 30	obese	5–9	0.22 (0.17–0.27)

Source: Guidelines on Maternal Nutrition in Iran [26] adopted from Institute of Medicine, 2009

Although the underlying mechanisms of the GWG have not yet been well explained [1], the results of various studies have shown that nonoptimal GWG, whether insufficient or excessive, is associated with a number of complications for both mother and infant. For example, women who gain less weight than the IOM recommendations are more likely to experience preterm labor and will have low birth weight infants [1]. In contrast, women who gain more weight than medical recommendations may face pregnancy complications such as preeclampsia, gestational diabetes, as well as complications, travail, and cesarean Sect. [5–7]. Non-optimal weight gain during pregnancy affects the pregnancy outcomes and has a negative impact on weight throughout life [8, 9].

While excessive GWG is a major concern in developed countries, malnutrition and inadequate GWG are common among women in low- and middle-income countries [10, 11]. Based on the results of studies, more than half of American women have excessive GWG. However, the Asian women have the highest prevalence of inadequate GWG (31%) [12]. Iran, as one of the developing countries in Southwest Asia, is no exception to this rule. For example, a study reported that 45.9% of Iranian women do not succeed to gain normalweight during pregnancy [13].

The IOM acknowledged that in addition to biology and individual behaviors, environmental factors also have a significant impact on GWG, and called for studies to be conducted to extensively examine social, cultural, and environmental contexts that influence GWG [4]. The results of research on social and environmental determinants suggest that residential area is associated with both inadequate GWG and excessive GWG [9, 14, 15]. Nevertheless, so far, few studies have examined the relationship between GWG and residential area. For example, in a study conducted in neighborhoods that were more likely to be socioeconomically disadvantaged, the relationship between poor neighborhood and gestational weight gain and gestational weight loss was examined [9]. In another study conducted in 2017, the relationship between residence in high-violence neighborhoods and GWG was assessed [15]. However, according to our search in various databases, so far no study has examined the status of GWG in the slums areas of Iran. The word “slum” is often defined as an informal residence in the outer parts of a city or even in the inner parts of a city [16]. Most slum dwellers are people who migrate to the big cities in the hope of finding a job and better financial means. They settle in insecure homes (such as windowless, earthen floor, leaky walls) or in unstable structures that are not strong enough against floods and earthquakes [17]. Poverty, lack of facilities and infrastructure, lack of access to safe drinking water resources, high density, limited access to health services, malnutrition and exposure to infectious diseases are the most important characteristics of residents of slums areas, which has created complex conditions in terms of access to health indicators for its residents [18–20]. Nearly one billion people worldwide now live in slum areas [16]. In Iran, the trend of living in this areas is growing due to the speed of urbanization [21]. Hamadan, which is one of the cities located in the mountainous region of western Iran, has many slums areas; according to the latest census conducted in 2017, 39% of its inhabitants reside in slums areas [17]. Thus, based on the above studies, it seems that conducting research in this regard would help healthcare policymakers to enhance the health level of pregnant women living in these regions. In addition, most available studies have evaluated women's weight gain status only at two time points (pre-pregnancy weight and postpartum weight) [9, 15] and are therefore unable to detect different patterns of GWG over weeks of pregnancy (1–41 weeks) and in turn different trimesters of pregnancy. Thus, in this study, we tried to determine the trend of GWG changes during the weeks of pregnancy in the suburban women of Hamadan using the multilevel ordinal model, which takes into account the longitudinal structure of the data.

## Materials and methods

### Study population and data collection

In this longitudinal study, the study sample consisted of pregnant women who referred to health care clinics in the slum areas of Hamadan in Iran from February 2021 to February 2022 and their information was recorded in “sib” (an abbreviation for the Persian equivalent of “integrated health system”). In the Sib system, which is a comprehensive electronic system, all information related to Iranian households and the type of health services required is recorded [22]. The number of slum centers in the present study was nine health care clinics and in each clinic, the information of all pregnant women was examined. We excluded from this sample women who had twins, as well as those whose weight information was not recorded. Gestational age was determined based on the date of the last menstrual period and was confirmed by ultrasound. All weight measurements were performed by experienced midwives using a digital smart scale with an accuracy of 100 g. The information related to 676 pregnant women was recorded, of which 167 values were missing, so these missing observations were removed from the total data with listwise form, and 509 pregnant women who were followed up during the weeks of pregnancy were analyzed. The number of visits of these 509 pregnant women was 1663 times in total.

### Predictive variables

Predictor variables were considered based on the literature and in the form of socio-economic variables, maternal characteristics, and an environmental variable. Variables of age, level of education (less than diploma (less than upper secondary education) / diploma (completion of upper secondary education) / higher than diploma (higher than upper secondary education)), maternal-employment status (housekeeper, other), husband employment status (self-employed / unemployed / worker / employee), and health insurance status (yes / no) were considered as socio-economic predictors. Based on definition the Merriam-Webster Dictionary, a housekeeper is a “member of a household who manages the domestic duties of the household”, a worker is “a person who does a particular job to earn money.” Whereas, an employee is “a person who works for another person or for a company for wages or a salary [23]”.

Variables of pre-pregnancy BMI (kg/m^2^), height, number of previous live child, history of abortion (yes / no), and planned pregnancy (yes /no) constituted the maternal characteristics. Season of conception (spring / summer / autumn or winter) was also considered as an environmental factor.

### Weight measurement before and during pregnancy

According to the care protocols of the Ministry of Health of Iran, in this study the visit times refers to the pre-pregnancy time and time when a pregnant woman comes for prenatal care, defined as weeks 6–10, 11–15, 16–20, 21–25, 26–30, 31–34, 35–37, 38, 39, and 40 [24]. These visit times can be divided according to the trimesters of pregnancy as follows: the first trimester of pregnancy (weeks 1–13), the second trimester of pregnancy (weeks 14–26), and the third trimester of pregnancy (weeks 27–40) [25].

The best criterion for determining the appropriate weight gain range for women during pregnancy is the use of BMI based on pre-pregnancy weight. If pre-pregnancy weight is not recorded, the pregnant woman's weight at the first visit (during the first 13 weeks of pregnancy) is considered as the initial weight of the pregnancy, provided that she does not have severe weight loss due to pregnancy nausea and vomiting [26].

In this study, pre-pregnancy BMI was calculated by respondents' height and weight before pregnancy and classified into four groups according to WHO standard criteria (Table 1) [27]. Also, the allowable total weight gain range and rates of weight gain from the beginning of week 13 onwards according to the IOM recommendation is presented in this table [28]. Women with GWG under the recommended range are considered as inadequate weight gain, those with GWG within the recommended range are regarded as adequate weight gain, and women with GWG above the recommended range are considered as excessive weight gain [29].

### Statistical methods and software

Samples were described using appropriate descriptive statistics. In order to compare the characteristics of pregnant women in different categories of pre-pregnancy BMI, analysis of variance test for quantitative variables and Chi-square test for qualitative variables were used. Also, in order to determine the factors affecting GWG and the trend of its changes during the weeks of pregnancy, a multilevel ordinal logistic regression model was used. All tests were performed at a significance level of 0.05.

### Multilevel ordinal logistic regression model

Multilevel models have been extensively developed for longitudinal data analysis. The simplest structure in a multi-level model is a 2-level model. In 2-level models, repetitive observations (level-1) are nested in individuals (level-2). In the present study, repeated measures included the weight of pregnant women during the weeks of pregnancy (level-1); these measures were nested within women (level-2). A 2-level ordinal logistics model with the random intercept is shown as follows:

1 $$logit\left[P\left({Y}_{ij}\le c\right)\right]={\alpha }_{c}+{x}_{ij}^{^{\prime}}\beta +{u}_{0i} ;\mathrm{c}=1.2.\dots .\mathrm{C}-1$$

where ${\alpha }_{c}$ is the C-1 intercept for the C ordinal model, ${x}_{ij}$’ s represent explanatory variables related to fixed effects, and ${u}_{0i}$ is the random intercept, which is assumed to have a normal distribution. In the present study, ${Y}_{ij}$ represents the outcome which is considered as inadequate, normal, and excessive weight gain respectively (C = 3). In this representation, a positive value of the beta coefficient indicates that higher values of the related explanatory variable are less associated with a higher probability of being in the GWG groups. Model 1 is called the proportional odds model since the effect of each factor (β) is the same across all outcome classes. However, sometimes it is possible that in one study the effect of some factors is the same among the outcome categories, but the effect of other factors changes between the outcome categories, which in this case the partial proportional odds model is used:

2 $$logit\left[P\left({Y}_{ij}\le c\right)\right]={\alpha }_{c}+{x}_{ij}^{^{\prime}}\beta +{({x}_{ij}^{*})}^{^{\prime}}{\beta }_{c}+{u}_{0i} ; \mathrm{c}=1.2.\dots .\mathrm{C}-1$$

In the above model, the ${x}_{ij}$ effect is the same across all categories, but the ${x}_{ij}^{*}$ effect changes as the categories change [30]. In order to find the best model from among the proportional and partial proportional odds models, a model can be selected that has the lowest Akaike information criterion (AIC).

### Software

In order to describe the data as well as to compare the characteristics of individuals in different categories of pre-pregnancy BMI, SPSS software version 24 was used. Also to fit the multilevel ordinal logistic regression model, the clmm2 function in the ordinal package [31] in R4.0.3 software was employed.

## Results

According to the results of Table 2, among 509 pregnant women based on pre-pregnancy BMI, 19 (3.7%) subjects were underweight, 202 (39.7%) subjects were normal weight, and 192 (37.7%) subjects were overweight, with 96 (18.9%) subjects being obese. The mean age of all participants was 28.54 ± 6.01 years. Most of the subjects, i.e. 361 (71.1%), were within the age range of 20–34 years where the mean age of overweight and obese women was significantly higher than that of the other two groups (*P*-value < 0.001). More than half of pregnant women (52.3%) had less than diploma education and a very large percentage of them (81.5%) were housewives About half of all pregnancies (47.9%) have occurred in spring. Groups of weight were significantly different in terms of planned pregnancy status (*P*-value = 0.001); underweight women had the highest percentage (73.7%) of unplanned pregnancies. Among the total participants, 78.2% had no underlying disease.

**Table 2:** Demographical feature and status of 509 pregnant women in pre-pregnancy time at 2021–2022

Quantitative features		Under weight (Mean ± SD)	Normal weight (Mean ± SD)	Over weight (Mean ± SD)	Obese (Mean ± SD)	Test Statistic	*p*-value
**maternal age (year)**		25.09** ± **5.55	27.48** ± **6.02	29.67** ± **5.57	29.19** ± **6.35	7.093	< 0.001*
**Pre-pregnancy BMI (kg/m**^**2**^**)**		17.19 ± 0.943	22.54 ± 1.73	27.35 ± 1.50	32.61 ± 2.46	876.180	< 0.001*
**Height (cm)**		162.42 ± 4.38	160.41 ± 6.06	160.97 ± 6.30	160.59 ± 6.19	0.779	0.506
**Qualitative features**		**Count (%)**					***p*-value**
		**Under weight (*n* = 19)**	**Normal weight (*n* = 202)**	**Over weight (*n* = 192)**	**Obese (*n* = 96)**	**Total**	
**Level of education**	**< diploma**	12(63.2)	109(54.0)	92(47.9)	53(55.2)	266(52.3)	0.102
	**diploma**	3(15.8)	62(30.7)	57(29.7)	34(35.4)	156(30.6)	
	**> diploma**	4(21.1)	31(15.3)	43(22.4)	9(9.4)	87(17.1)	
**Season of conception**	**Spring**	7(36.8)	92(45.5)	96(50.0)	49(51.0)	244(47.9)	0.797
	**Summer**	10(52.6)	79(39.1)	69(35.9)	33(34.4)	191(37.5)	
	**Autumn and winter**	2(10.5)	31(15.3)	27(14.1)	14(14.6)	74(14.5)	
**Maternal-Employment Status**	**Housekeeper**	15(78.9)	157(77.7)	163(84.9)	80(83.3)	415(81.5)	0.298
	**Non House keeper**	4(21.1)	45(22.3)	29(15.1)	16(16.7)	94(18.5)	
**Husband Employment Status**	**Self-employed**	11(57.9)	124(61.4)	116(60.4)	64(66.7)	315(61.9)	0.160
	**Unemployed**	4(21.1)	21(10.4)	8(4.2)	6(6.3)	39(7.7)	
	**Worker**	3(15.8)	43(21.3)	51(26.6)	19(19.8)	116(22.8)	
	**Employee**	1(5.3)	14(6.9)	17(8.9)	7(7.3)	39(7.7)	
**Number of previous live child**	**Zero**	10(52.6)	93(46.0)	62(32.3)	27(28.1)	192(37.7)	0.002*
	**One**	8(42.1)	85(42.1)	84(43.8)	44(45.8)	221(43.4)	
	**Two or more**	1(5.3)	24(11.9)	46(24.0)	25(26.0)	96(18.9)	
**History of abortion**	**Yes**	0(0.00)	13(6.4)	16(8.3)	5(5.2)	34(6.7)	0.465
	**No**	19(100)	189(93.6)	176(91.7)	91(94.8)	475(93.3)	
**Planned pregnancy**	**Yes**	5(26.3)	85(42.1)	109(56.8)	57(59.4)	256(50.3)	0.001*
	**No**	14(73.7)	117(57.9)	83(43.2)	39(40.6)	253(49.7)	
**Health insurance status**	**Yes**	16(84.2)	182(90.1)	169(88.0)	87(90.6)	454(89.2)	0.768
	**No**	3(15.8)	20(9.9)	23(12.0)	9(9.4)	55(10.8)	
**Underlying disease**	**Yes**	3(15.8)	43(21.3)	41(21.4)	24(25.0)	111(21.8)	0.793
	**No**	16(84.2)	159(78.7)	151(78.6)	72(75.0)	398(78.2)	

^*^ significant test in level 0.05

The results presented in Table 3 are based on data from 1663 visits from 509 pregnant women; during each trimester, a woman may has visited several times. According to the results of this table, 594 pregnant women referred in the first trimester, 825 subjects in the second trimester, and 244 subjects in the third trimester. A very large percentage of subjects (85.4%) in the first trimester and nearly half of the subjects (49.1%) in the second trimester did not have adequate weight gain, but in the third trimester, most women (38.9%) gained adequate weight or (36.5%) gained excessive weight.

**Table 3:** Weight gain status across time by group: response proportions and sample sizes

Timpoint of visit			
Weight gain	**First trimester**	**Second trimester**	**Third trimester**
Inadequate weight gain	507(85.4)	405(49.1)	60(24.6)
Adequate weight gain	29(4.9)	273(33.1)	95(38.9)
Excessive weight gain	58(9.8)	147(17.8)	89(36.5)
N	594	825	244

According to the AIC, the multilevel ordinal model with partial proportional odds in which the effect of the predictor of the week of visit has not been the same among cumulative logits, was a more appropriate model (with less AIC) to identify the factors affecting GWG than the model with proportional odds (AIC = 2495.121, 2603.546, respectively). The results of its fitting are presented in Table 4. Since the outcome under consideration (GWG) had three categories (inadequate weight gain, adequate weight gain, and excessive weight gain), the ordinal model was expressed using two cumulative logit models. The first cumulative logit model expresses the odds of inadequate weight gain versus adequate and excessive weight gain (group 1 vs. groups 2 and 3). The second cumulative logit model expresses the odds of excessive weight gain versus inadequate and adequate weight gain (group 3 vs. groups 1 and 2).

**Table 4:** Estimates for parameters of partial-proportional odds model

Variable	Partial-proportional				*P*-value
	**(Inadequate | Adequate)**^**a**^		**(Adequate | Excessive)**^**b**^		
	**Estimate (SE)**	**OR** (95% CI)	**Estimate (SE)**	**OR** (95% CI)	
**Week of visit**	-0.238(0.014)	0.788(0.766,0.810)	0.110(0.011)	1.116(1.09, 1.140)	-
**Pre-pregnancy BMI**	0.068(0.029)	1.070(1.011, 1.133)	-0.068(0.029)	0.934(0.883,0.989)	0.021*
**Maternal age**	0.030(0.025)	1.030(0.981, 1.082)	-0.030(0.025)	0.971(0.924, 1.019)	0.239
**Maternal education (Lower than diploma as reference)**					
Diploma	-0.070(0.290)	0.932(0.528,1.646)	0.070(0.290)	1.073(0.607, 1.893)	0.809
College grad	-0.364(0.374)	0.694(0.334,1.446)	0.364(0.374)	1.440(0.691, 2.995)	0.330
**Number of previous live births (Zero as reference)**					
One	0.128(0.423)	1.137(0.496,2.60)	-0.128(0.423)	0.879(0.384, 2.016)	0.762
Two or more	-0.026(0.515)	0.974(0.355,2.673)	0.026(0.515)	1.026(0.374, 2.816)	0.959
**Planned pregnancy (Yes as reference)**					
No	0.505(0.410)	1.658(0.742, 3.70)	-0.505(0.410)	0.603(0.270, 1.348)	0.218
**Underlying disease (No as reference)**					
Yes	-0.543(0.312)	0.581(0.315,1.071)	0.543(0.312)	1.722(0.934, 3.172)	0.081
**Season of conception (Spring as reference)**					
Summer	-0.015(0.286)	0.985(0.562, 1.725)	0.015(0.286)	1.015(0.579, 1.778)	0.959
Autumn & winter	-0.727(0.460)	0.483(0.196, 1.191)	0.727(0.460)	2.070(0.839, 5.097)	0.114
**Husband Employment-Status (Self-employed as reference)**					
Unemployed	-0.729(0.503)	0.482(0.180, 1.293)	0.729(0.503)	2.073(0.773, 5.556)	0.147
Worker	-0.059(0.311)	0.943(0.512, 1.734)	0.059(0.311)	1.061(0.576, 1.951)	0.849
Employee	0.291(0.483)	1.334(0.519, 3.448)	-0.291(0.483)	0.747(0.290, 1.926)	0.547
**Random components of partial -proportional odds**					
Intercept sd	2.314				

*SE* Standard error

* indicates *p* < 0.05, 95% CI indicates 95% confidence interval for OR

^a^ logit comparing inadequate weight gain vs. adequate and excessive weight gain

^b^ logit comparing excessive weight gain vs. inadequate and adequate weight gain

Based on the results of fitting multi-level ordinal models reported in Table 4, pre- pregnancy BMI has a significant effect on the odds of inadequate GWG (*P*-value = 0.021); with one unit increase in pre-pregnancy BMI, the odds of inadequate GWG grows by 1.07 times compared to adequate and excessive GWG. Although the odds of excessive weight gain during pregnancy in women who become pregnant in the autumn and winter are almost twice the odds in mothers who become pregnant in the spring, but the Wald test showed that this relationship was not statistically significant (OR = 2.070; 95% CI: 0.839, 5.097; *P*-value > 0.05). Also, based on the sample information in the present study, the odds of inadequate weight gain in pregnant women with unplanned pregnancies is 1.685 times higher than women with planned pregnancies, but this relationship was not statistically significant (OR = 1.685; 95% CI: 0.742, 3.70; *P*-value > 0.05). For other variables, OR’s with confidence intervals is reported in the Table 4.

**Fig. 1:**
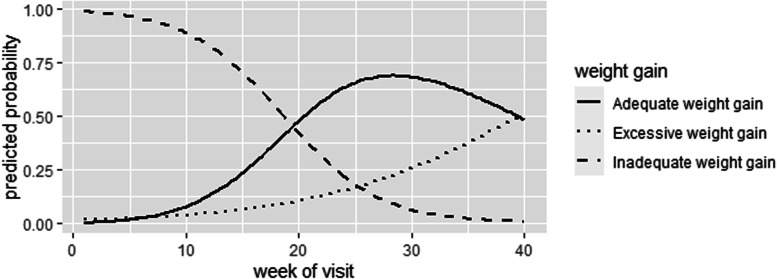
Graph of the pattern of changes in GWG probability (inadequate, adequate, and excessive weight gain) during the weeks of pregnancy (weeks 1–40)

Figure 1 displays the pattern of GWG probability changes obtained from a multilevel ordinal model with partial proportional odds. According to the results of Fig. 1, up to the 19th week of pregnancy (middle of second trimester), the probability of inadequate weight gain is higher than that of adequate and excessive weight gain. However, after the 19th week of pregnancy, the probability of adequate weight gain rises while the probability of inadequate weight gain decreases, where the excessive weight gain grows with a moderate slope. At 25–30 weeks of pregnancy, the probability of adequate weight gain is located at the highest point. From week 30 onwards, the probability of excessive weight gain rises with a steeper slope, and the trend of the probability of inadequate weight gain continues to decline.

In general, as the number of weeks of pregnancy increased, the probability that pregnant women experience inadequate weight gain decreased in this study. By the 30th weeks, women are more likely to gain adequate weight, but from the 30th week onwards, the probability of adequate weight gain gradually diminishes compared to previous weeks and the probability of excessive weight gain increases, to the extent that in the last week of pregnancy these two graphs become almost equal.

## Discussion

Weight gain during pregnancy is an influential factor in maternal and fetal health [15]. Pregnancy care, including monitoring pregnancy weight in slums communities, is faces with many challenges due to poverty and lack of facilities as well as lack of access to health care [32]. Thus, due to the importance of this issue, in this study we tried to identify the factors affecting GWG in women in slum areas of Hamadan as well as to determine the general trend of its changes during the weeks of pregnancy. Note that based on the review of texts, no study has been conducted on this issue in the slums of Iran. According to the results of this study, a little more than half of pregnant women (56.6%) were overweight or obese based on pre-pregnancy BMI and most of them did not gain normal weight in the first and second trimesters. Perhaps one of the most important factors related to overweight or obese before pregnancy is inactivity among women residing in slum areas [33]. The women living in slum areas do not have adequate physical activity due to lack of facilities and suitable places for physical activity, lack of necessary support from relevant organizations, as well as traditional attitudes about women's physical activity. On the other hand, it seems that one of the causes of overweight in slum areas is their type of diet. In poor areas, due to high protein costs and lack of access to fresh foods, consumption of carbohydrate and fat is high [34, 35]. Also, from the results of studies, it can be found that carbohydrate consumption is positively associated with obesity [36]. Nevertheless, although most people were overweight or obese before pregnancy, a very large percentage of people (85.4%) in the first trimester as well as about half of the people (49.1%) in the second trimester did not gain adequate weight. According to the results of Fig. 1, up to the 19th week of pregnancy (middle of second trimester), the probability of inadequate weight gain has been higher than that of adequate and excessive weight gain. A study conducted in 2014 also shows that women residing in socio-economically disadvantaged neighborhoods gain inadequate weight during pregnancy [9]. This is because living in poor areas affects the health of pregnant women through lack of access to healthy food options, limited health care options [9], and exposure to stressors such as violence and crime [15] that occur abundantly in these neighborhoods. On the other hand, according to the results of other studies, when pre-pregnancy BMI increases, GWG decreases, both in general and in trimesters [37]. In our study, since most people were overweight or obese before pregnancy, this has not been very unexpected. Also, one of the causes of inadequate weight gain in the first trimester can be attributed to morning sickness. Nausea and vomiting are common pregnancy experiences that affect approximately two-thirds of pregnant women and are symptoms of the first trimester of pregnancy [38]. Meanwhile, there is evidence from previous studies suggesting that weight gain in the second trimester and / or third trimester strongly affects the birth weight and infant growth. For example, a prospective cohort study found that although GWG is positively correlated with birth weight in all three trimesters, the second trimester has the most impact [7]; weight loss in this trimester will cause the birth of low-birth-weight infants [39]. Thus, effective interventions to compensate for GWG in the second trimester in these areas should be given high priority by health officials. The study showed that, although women who became pregnant in the autumn and winter had a higher odds for experience excessive weight gain during pregnancy, but it was not statistically significant. The study conducted in rural Bangladesh also had similar results [10]. This is because these people spend the second and third trimesters of their pregnancies in the spring and summer, when access to food resources is greater [10]. Since the majority of women in the present study had a diploma or lower education level and in terms of employment they were housewives, creating appropriate strategies can be effective in improving the health literacy of households in these areas. The strength of the present study has been the availability of pre-pregnancy BMI data (strong predictor of GWG) in most participants (91%) as well as the evaluation of GWG in all weeks of pregnancy, and of course use of multi-level models that are accurate in outcome prediction [40]. However, one of the limitations of our study was the lack of access to information on nutritional factors of pregnant women residing in slum areas. Also, since dietary habits before and during pregnancy have a potential effect on GWG and maternal and fetal health [41], it is suggested that the effect of these factors be also examined in future studies. It is also suggested that the relationship between trimesters specific GWG with the weight of infants born be examined in these areas. Future studies can also compare GWG rates in women living in slum areas with areas with a higher socio-economic level.

## Conclusion

Recommendations related to normal weight gain during pregnancy is one of the basic measures in the care of this period, which should be provided at the first visit and after confirmation of the mother's pregnancy. It is also necessary to monitor the mother's weight gain under the supervision of health center Employees. According to the results of this study, most people were overweight and obese before pregnancy and women's weight gain in the first and second trimesters was inadequate. Therefore, it seems necessary that pregnant mothers in slums areas who are economically and socially poor and do not have enough access to health care centers should be identified and covered by support institutions.

## Data Availability

The data set analyzed during the present study is not available to the public because it belongs to the “sib” system and has a limited use license, but is available at the reasonable request of the corresponding author.
